# Superomedial partial arytenoidectomy for voice improvement by correction of posterior glottic insufficiency

**DOI:** 10.1007/s00405-020-05859-2

**Published:** 2020-02-18

**Authors:** Rutger Mahieu, Derrek Heuveling, Hans Mahieu

**Affiliations:** 1 Department of Otorhinolaryngology, University Medical Center Utrecht, University of Utrecht, Heidelberglaan 100, 3584 CX Utrecht, The Netherlands; 2 Department of Otorhinolaryngology, Meander Medical Center, Amersfoort, The Netherlands

**Keywords:** Partial arytenoidectomy, Glottic insufficiency, Dysphonia, Voice improvement

## Abstract

**Purpose:**

Arytenoid resection is a well-known intervention to improve glottic airway. Superomedial partial arytenoidectomy (SPA) can also be used for voice improvement by correcting posterior glottic insufficiency in patients with an obstructing anteromedially prolapsed arytenoid. Posterior glottic insufficiency can be difficult to address and traditionally involves challenging arytenoid repositioning procedures. This study aimed to compare postoperative functional voice outcomes in patients who underwent SPA to pre-operative voice status. Second, consequences of concomitant injection augmentation in patients who underwent SPA were studied. Additionally, presenting the surgical technique.

**Methods:**

In this retrospective cohort study, pre-operative and postoperative clinical data of patients who underwent SPA between 2004 and 2018 were analyzed. Both short- and long-term voice outcomes were assessed using Voice Handicap Index (VHI) and maximum phonation time (MPT). Pre- to postoperative assessment changes (delta: *δ*) were applied to multivariate analyses.

**Results:**

A total of 105 patients were included, of which 91 had hemilaryngeal immobility, 25 had undergone previous phonosurgical procedures and 45 received concomitant injection augmentation. Patients who underwent SPA had significant improvement of VHI and MPT. In 81% of our population, laryngeal framework surgery was avoided. Multivariate analyses showed significantly improved short-term voice outcomes in patients who received injection augmentation concomitantly to SPA. Finally, *δ*MPT was a significant predicting factor regarding additional procedures in patients who underwent SPA.

**Conclusion:**

SPA is a safe and efficient procedure for voice improvement in patients with posterior glottic insufficiency due to an obstructing anteromedially prolapsed arytenoid. We recommend performing this procedure combined with injection augmentation.

## Introduction

Partial or complete arytenoidectomy is a well-known intervention to improve glottic airway in patients with bilateral vocal fold immobility [1–6].

**Fig. 1:**
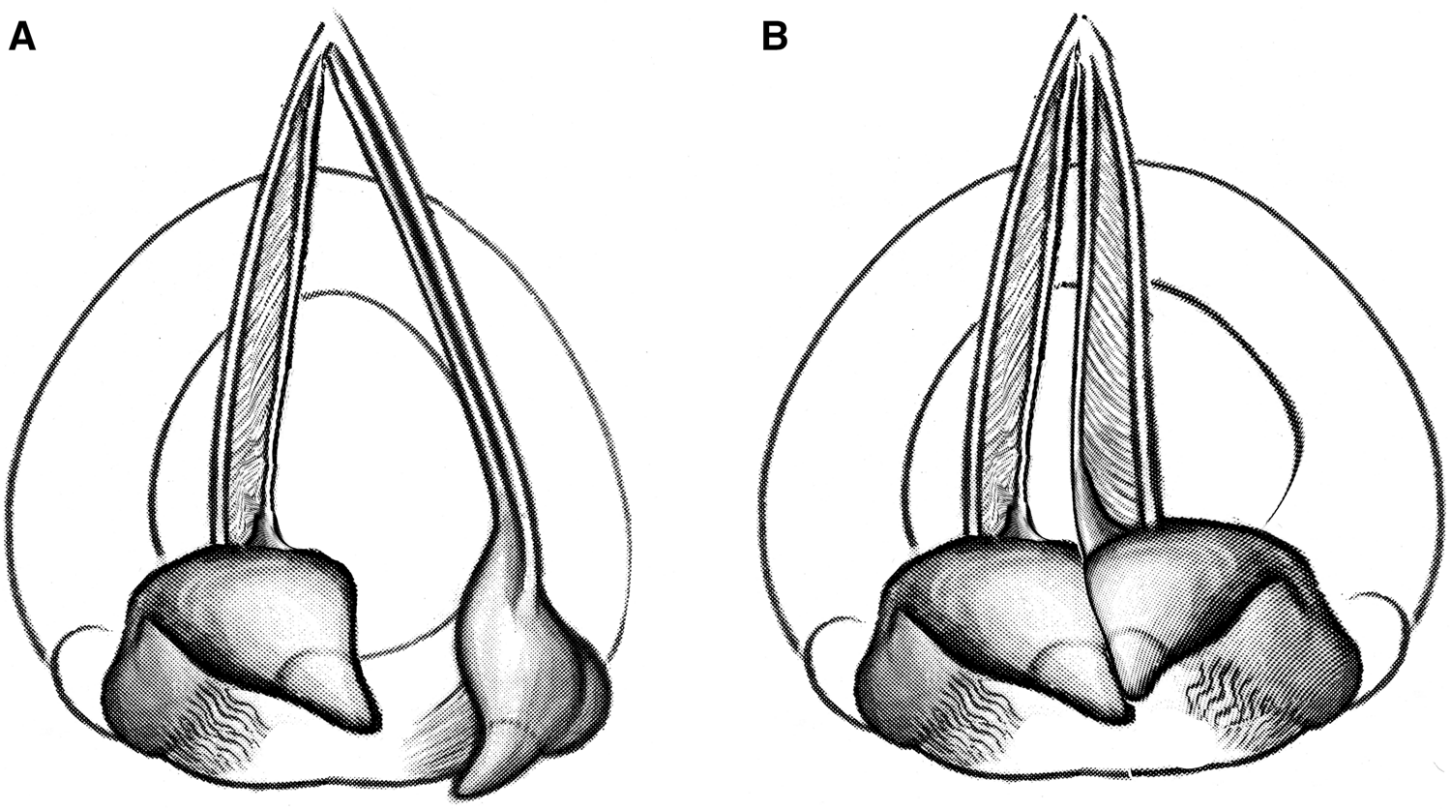
Schematic representation of obstructing anteromedially prolapsed left arytenoid in hemilaryngeal immobility. **a** During respiration. **b** During phonation; posterior incomplete glottis closure due to obstruction of the left arytenoid

However, a similar procedure can also be used for voice improvement by correcting posterior glottic insufficiency. In many patients with hemilaryngeal immobility, an anteromedially prolapsed arytenoid on the immobile side can be observed, blocking the contralateral mobile arytenoid, thus causing a supraglottic obstruction and precluding adequate posterior glottic closure (Fig. 1).

Occasionally, similar mechanisms can be observed in patients with normal bilateral laryngeal mobility with medially protruding arytenoids, causing both arytenoids to collide, whereas both vocal processes remain separated, resulting in posterior glottic insufficiency.

Removal of the obstructing structure, i.e. the arytenoid’s superomedial part, may facilitate contralateral compensation resulting in improval of glottic closure. Accordingly, patients with insufficient posterior glottic closure due to an obstructing arytenoid are amenable for superomedial partial arytenoidectomy (SPA). It is, however, important to realize that complete contralateral hemilaryngeal compensation cannot be guaranteed and may take time to develop.

To our knowledge, only one previous SPA case, with the intention to improve the voice, in a patient with unilateral hemilaryngeal immobility has been reported in English literature [7]. This case report mentions having learned about SPA from senior author HM.

In the present retrospective study, a series of 105 patients with posterior glottic insufficiency due to arytenoid obstruction, who underwent SPA for voice improvement, is presented.

The primary aim of this study was to compare postoperative functional voice outcomes in patients who underwent SPA, in terms of Voice Handicap Index (VHI) and maximum phonation time (MPT), to pre-operative voice status. Second, consequences of concomitant injection augmentation regarding short- and long-term voice outcomes were studied.

## Materials and methods

This retrospective study was conducted according to the principles of the Declaration of Helsinki and in accordance with the Medical Treatment Agreement Act and the General Data Protection Regulation.

### Patients

Patients with dysphonia caused by insufficient posterior glottic closure due to an anteromedially prolapsed obstructing arytenoid were considered amenable for SPA and, consequently, included in this study. Patients with other indications than primarily vocal complaints (e.g. airway compromise) and patients with progressive neurological or neuromuscular disease were excluded from this study. The remaining exclusion criterium was the lack of follow-up data.

Pre-operative and postoperative clinical data of patients who underwent SPA, treated by senior author HM, between April 2004 and May 2018 at the Department of Otorhinolaryngology, Meander Medical Center Amersfoort, the Netherlands, a tertiary laryngological referral center, were analyzed.

Initially, only SPA was performed, but since 2011, patients with hemilaryngeal immobility underwent concomitant injection augmentation. In patients with a high risk for airway compromise, concomitant injection augmentation was not deemed feasible. Therefore, in these patients, SPA was performed as the sole procedure.

Out of a total of 115 patients who underwent SPA, 105 patients met the inclusion criteria for this study. Patients were excluded on basis of progressive neurological disease (*n* = 3) and the lack of postoperative voice outcomes (*n* = 7).

Most of the included patients were referred to undergo (comprehensive) laryngeal framework surgery. However, after laryngostroboscopic examination, they were considered amenable for SPA. In all patients, injection augmentation, laryngeal framework surgery and SPA were offered, after which patients were engaged for participation in making the decision.

### Surgical technique

The procedure was performed during microlaryngoscopy under general anesthesia. The larynx was exposed using a Remacle Pototschnig laryngoscope^®^ (Richard Wolf GmbH) or, if more difficult to expose, a Benjamin-Parsons Slimline Laryngoscope^®^ (Storz GmbH). All laser-safety regulations including oxygen levels and laser-safe tubes were adhered to.

**Fig. 2:**
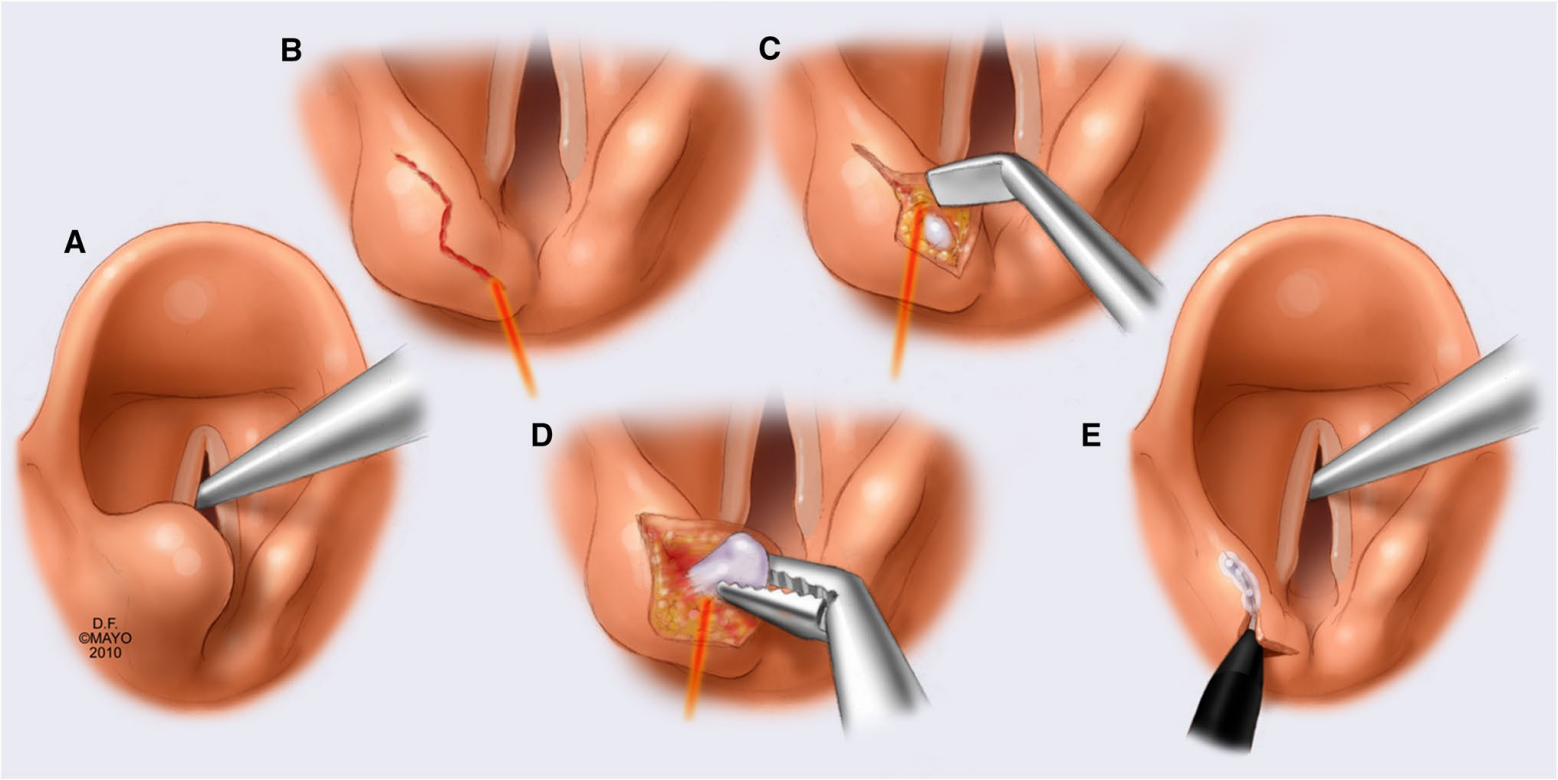
Surgical technique of superomedial partial arytenoidectomy. Used with permission of Mayo Foundation for Medical Education and Research. **a** Anteromedially prolapsed left arytenoid in hemilaryngeal immobility. **b** Mucosal incision over the concerning arytenoid. **c** Developing small mucosal flaps by laser, for further exploration. **d** Removing the accessory cartilages with blunt dissection and laser vaporization. **e** The mucosal flap is replaced and sealed

Using a Lumenis 30C^®^ CO_2_ laser with Acu-spot (Energy 100 mJ; power settings 1.5 W superpulse in continuous mode), a Z-shaped (left arytenoid) or S-shaped (right arytenoid) mucosal incision over the obstructing arytenoid was made (Fig. 2b). Small mucosal flaps were developed by laser from this incision, for further exploration (Fig. 2c). Subsequently, the accessory cartilages (cuneiform and corniculate) were removed using a combination of blunt dissection and laser vaporization (Fig. 2d). Then, the arytenoid’s superomedial part was exposed and vaporized, almost until the vocal process level. Care needs to be taken not to infringe the crico-arytenoid joint or vocal process. Preservation of these structures, including mucosal flaps, arytenoid’s full base and all muscular attachments, is pertinent.

The mucosal flaps were redraped intermittently to confirm that enough cartilage was removed.

Finally, the mucosal flaps were replaced, closing the mucosal defect, and sealed using TISSEEL^®^ (Baxter Healthcare Corporation) two-component fibrin sealant to reduce the risk of granuloma (Fig. 2e). All in all, SPA requires approximately 20–30 min to perform.

In exceptional cases, SPA was performed contralaterally of the hemilaryngeal immobile side (*n* = 3), since the contralateral arytenoid’s suprastructure seemed to cause the obstruction.

### Injection augmentation

Since 2011, concomitant injection augmentation was performed in all patients who underwent SPA, except in those with high risk for airway compromise.

Injection augmentation was performed simultaneously with SPA as a single-stage procedure; under general anesthesia, transorally under direct visualization using a laryngoscope, microscope and/or 70° angled rigid telescope.

Injection agents used were calcium hydroxyapatite (Radiesse Voice^®^, Merz Pharmaceuticals GmbH and RenuVoice^®^, Regenscientific Corporation) or hyaluronic acid (Esthelis^®^, Merz Anteis S.A GmbH and Juvederm^®^, Allergan Corporation). The amount of injected material ranged from 0.3 to 0.7 cc.

### Functional assessment of voice

Voice assessments were acquired pre-operatively, within 6 months postoperatively, more than 6 months, but within 12 months postoperatively and during the last outpatient visit.

The level of self-assessed voice impairment was measured using VHI-30, a world-wide used standardized and validated questionnaire [8]. It has been translated into Dutch and validated in previous studies [9–11]. VHI-30 consists of 30 statements on voice-related aspects in daily life, with 5 response levels, scored 0–4, leading to a total VHI score, ranging from 0 to 120. Higher scores correspond with worse voice-related functional status [10].

Additionally, the maximum phonation time (MPT), as a simple indicator of glottis closure, was documented of each patient. MPT, consisted of the longest period of time while phonating /a/ in three efforts.

Differences between pre- and postoperative voice outcomes were represented as δ-variables accordingly (e.g. δMPT, δVHI).

### Sample size

To assess whether reliable statistical judgements can be made from our collected data, with the intent to compare postoperative functional voice outcomes in patients who underwent SPA to pre-operative voice status, a sample size calculation was conducted. This study’s sample size calculation is based on van Gogh et al. [10], where a group sample size of 36 achieves 90% power (*β* = 0.9) to detect a mean difference of 15.0 in VHI, with estimated group standard deviation of 19.40 and significance level (*α*) of 0.05, using a two-sided sample *t* test. In conclusion, this study’s population should consist of at least 36 patients to enable reliable statistical judgments.

### Missing data

Missing data were handled by means of pairwise deletion (Little’s MCAR test; *p* = 0.685). Thus, minimizing biased estimates caused by missing data, while preserving sufficient power for analyses.

### Statistical analyses

All data were analyzed with professional statistics software (IBM SPSS Statistics Version 25.0.). Data were expressed as mean ± SD, unless otherwise indicated, for continuous variables. Number of cases and percentages were represented for categorical variables.

To compare pre- and postoperative voice outcomes in our population, univariate analysis using paired-sample t test was applied for parametric continuous variables, while Friedman’s two-way ANOVA and Mann Whitney *U* test were applied for non-parametric continuous variables.

For these analyses, pre-operative VHI and MPT were compared with postoperative VHI and MPT outcomes in pairs:

*(“Pair 1”)* Pre-operative VHI and MPT were compared with VHI < 6 months and MPT < 6 months, respectively.

*(“Pair 2”)* Pre-operative VHI and MPT were compared with VHI > 6 months and MPT > 6 months, respectively.

*(“Pair 3”)* Pre-operative VHI and MPT were compared with last VHI and MPT measured, respectively.

These analyses were also conducted separately for patients who only underwent SPA without concomitant injection augmentation. Furthermore, differences in pre- to postoperative voice outcomes (*δ*VHI, *δ*MPT), between SPA with concomitant injection augmentation and only SPA, were compared using independent-sample *t* test and Mann Whitney *U* test. Finally, for comparing complication rates concerning SPA with and without concomitant injection augmentation, *χ*^2^ test was used.

Multiple linear regression analyses were applied to assess independent correlates of *δ*VHI and δMPT outcomes in our population. Covariates were age, gender, hemilaryngeal immobility, cause of hemilaryngeal immobility, previous procedures, concomitant injection augmentation, pre-operative VHI, pre-operative MPT, larynxtrauma and radiotherapy/radio-chemotherapy in patient history.

To assess independent correlates of patients who underwent an additional procedure, binary logistic regression analysis was applied. Covariates were age, hemilaryngeal immobility, *δ*VHI, *δ*MPT, radiotherapy/radio-chemotherapy, larynxtrauma, concomitant injection augmentation, pre-operative MPT, pre-operative VHI and postoperative complications.

A *p* value of < 0.05 was regarded as statistically significant.

## Results

In this population of 105 included patients, 91 (87%) patients had hemilaryngeal immobility, 25 (24%) patients had undergone previous surgery for vocal problems, 45 (43%) consecutive patients received concomitant injection augmentation, 4 (4%) patients underwent SPA in 2 tempi, of which 1 contralateral. The mean interval between occurrence of hemilaryngeal immobility (if applicable) and SPA was 18 months (SD ± 95.4), ranging from 3 months until more than 50 years. Ten patients underwent SPA within 6 months of occurrence of hemilaryngeal immobility, three of whom were idiopathic. One of these three recovered mobility 3 months following SPA.

Population characteristics are displayed in Table 1.

**Table 1:** Patient characteristics

Variable	Included patients (*N* = 105)
AGE; MEAN (SD)	49.7 (± 16.6)
FEMALE (%)	59 (56%)
Side of hemilaryngeal immobility (%)	
Left	55 (53%)
Right	36 (34%)
Normal bilateral laryngeal mobility	14 (13%)
Cause of hemilaryngeal immobility (%)	
eci	24 (23%)
Neck-or thorax procedure	56 (53%)
Other	11 (11%)
Not applicable	14 (13%)
Arytenoid reduction left (%)	60 (57%)
Previous procedures (%)	
None	80 (75%)
Thyroplasty	6 (6%)
Thyroplasty + arytenoid adduction	1 (1%)
Microlaryngoscopical surgery	6 (6%)
Injection augmentation	6 (6%)
Multiple	6 (6%)
Concomitant injection augmentation (%)	
None	60 (57%)
Hyaluronic acid (Esthelis®. Juvederm®)	6 (6%)
Calcium hydroxyapatite (Radiesse®. Renu®)	39 (37%)
Comorbidity in patient history (%)	
Larynxtrauma	13 (12%)
RT/RTCHT	15 (14%)
Malignancy	24 (23%)
Congenital disorder	1 (1%)
CHNP-procedure	7 (7%)
Thyroid procedure	15 (14%)
Multiple	16 (15%)
Hospital stay in days; mean (SD)	1.1 (± 0.4)
Follow-up time in months; mean (SD)	19.4 (± 20.5)
Pre-operative vhi; mean (SD)	61.2 (± 19.4)
Pre-operative mpt in seconds; median (IQR)	6.0 (5)

*SD* standard deviation, *eci* e causa ignota, *RT* radiotherapy, *RTCHT* chemoradiation, *CHNP* cervical herniated nucleus pulposus, *VHI* Voice Handicap Index, *MPT* maximum phonation time, *IQR* interquartile range

### Voice outcome

Preoperative mean VHI was 61.2 (SD ± 19.4). Postoperative mean VHIs were < 6 months 39.3 (SD ± 19.5), > 6 months 38.6 (SD ± 22.0) and last measured within 12 months 37.6 (SD ± 21.8). VHI was improved in 86 of 98 cases.

Preoperative median MPT was 6.0 s (IQR 5.0). Postoperative median MPTs were < 6 months 9.0 s (IQR 7.0), > 6 months 10.0 s (IQR 8.0) and last measured within 12 months 10.5 s (IQR 8.0). MPT was improved in 86 of 101 cases.

Postoperative VHI and MPT (< 6 months postoperatively, > 6 months postoperatively and last measured postoperatively) were significantly improved as compared to pre-operative VHI and MPT (*p* < 0.001).

Table 2 shows that VHI and MPT outcomes were also significantly improved postoperatively in patients who only underwent SPA without concomitant injection augmentation (*p* < 0.001).

**Table 2:** Postoperative voice outcomes as compared to pre-operative voice status in patients who only underwent SPA

	Mean difference	SD	95% CI of difference	Sig.
VHI after SPA only^a^				
Pair 1				
VHI pre-operative—VHI < 6 months	15.89	± 17.60	[10.67–21.12]	< 0.001*
Pair 2				
VHI pre-operative—VHI > 6 months	19.86	± 18.64	[14.12–25.60]	< 0.001*
Pair 3				
VHI pre-operative—VHI	23.04	± 19.47	[17.67–28.40]	< 0.001*

	Median difference	Test statistic	Standard error	Sig.
MPT after SPA only^b^				
Pair 1				
MPT pre-operative—MPT < 6 months	3.00	− 1.53	0.33	< 0.001*
Pair 2				
MPT pre-operative—MPT > 6 months	4.00	− 1.52	0.33	< 0.001*
Pair 3				
MPT pre-operative—MPT	3.50	− 1.55	0.33	< 0.001*

Bold script with * indicates significant values

*SD* standard deviation, *CI* confidence interval, *Sig.* significance, *VHI* Voice Handicap Index, *SPA* superomedial partial arytenoidectomy, *MPT* maximum phonation time

^a^ Paired-sample *t* test

^b^Friedman’s two-way ANOVA

Table 3 shows that *δ*VHI < 6 months outcomes were significantly better in patients who underwent concomitant injection augmentation, as compared to patients who only underwent SPA (*p* = 0.001). However, δVHI > 6 months and δVHI outcomes were not significantly better in those who underwent concomitant injection augmentation, as compared to patients who only underwent SPA.

**Table 3:** Differences in δVHI-variables between patients who only underwent SPA and patients who underwent both SPA and concomitant injection augmentation

	Mean	Mean difference	95% CI of difference	Sig
δVHI < 6 months^a^				
Only SPA (n = 46)	15.89	14.85	[6.21 to 23.49]	0.001*
SPA + injection augmentation (n = 43)	30.74			
δVHI > 6 months^a^				
Only SPA (n = 43)	19.86	3.86	[− 4.40 to 12.12]	0.346
SPA + injection augmentation (n = 32)	23.72			
δVHI^a^				
Only SPA (n = 53)	20.28	6.90	[− 1.00 to 14.79]	0.086
SPA + injection augmentation (n = 45)	27.18			

Bold script with * indicates significant values

*VHI* Voice Handicap Index, *δ* difference between pre- and postoperative assessment, *SPA* superomedial partial arytenoidectomy, *SD* standard deviation, *CI* confidence interval, *Sig.* significance

^a^Independent samples *T* test

In addition, *δ*MPT < 6 months and δMPT outcomes were significantly better in patients who underwent concomitant injection augmentation, as compared to patients who only underwent SPA (*p* = 0.025; *p* = 0.004).

Multiple linear regression analysis with difference between pre- and postoperative VHI (δVHI) as the dependent variable, showed that only pre-operative VHI outcome correlates significantly with δVHI outcome measures (*p* = 0.006, *B* = 0.321). Ergo, higher pre-operative VHI outcome corresponds with greater improvement of postoperative VHI within 12 months after procedure.

Multiple linear regression analysis with difference between pre- and postoperative MPT (*δ*MPT) as the dependent variable, showed that concomitant injection augmentation significantly correlates with *δ*MPT and increases δMPT by 2.9 s (*p* = 0.007, *B* = 2.892). Additionally, multiple linear regression analyses with *δ*VHI < 6 months as the dependent variable, showed that both pre-operative VHI outcome and concomitant injection augmentation correlated positively with *δ*VHI < 6 months outcome (*p* < 0.001, *B* = 0.488; *p* = 0.036, *B* = 9.099). Hence, higher pre-operative VHI outcome and concomitant injection augmentation correspond with greater improvement of postoperative VHI within 6 months after procedure.

Finally, there was a positive correlation between concomitant injection augmentation and *δ*MPT < 6 months (*p* = 0.034, *B* = 1.794), meaning that concomitant injection augmentation corresponds with greater improvement of postoperative MPT within 6 months after the procedure.

### Complications

In this population, 13 patients (12%) presented with complications of which 5 (5%) required intervention (Clavien-Dindo Grade II/III [12]). Of these 13 patients, 5 patients developed a granuloma, of which 3 patients required no intervention (Clavien–Dindo Grade I). Furthermore, two patients presented with postoperative dyspnea and two patients presented with laryngeal edema, which recovered promptly and required no intervention (Clavien–Dindo Grade I). In addition, one patient had short-term temporary loss of sensibility of the tongue, which recovered spontaneously (Clavien–Dindo Grade I). Finally, in three cases, antibiotics were prescribed due to pneumonia (*n* = 1), local infection (*n* = 1) and prophylactic for short-term postoperative aspiration (*n* = 1) (Clavien–Dindo Grade II). A postoperative sore throat, not requiring analgetics, was not regarded as a complication and occurred in five patients.

Univariate analysis, by means of *χ*^2^ test, showed that there was no significant difference in complication rates between patients who underwent SPA with concomitant injection augmentation as compared to patients who underwent SPA as a sole procedure (*p* = 0.392).

### Additional procedures

Univariate analyses showed that 34 (32%) patients who underwent SPA requested an additional procedure after a mean follow-up time of 17.3 months (± SD 15.8). From these 34 patients who requested an additional procedure, 17 patients (50%) underwent laryngeal framework surgery, of which 4 required arytenoid adduction, 14 patients (41%) underwent additional injection augmentation and 3 patients (9%) underwent multiple procedures for voice improvement.

Binary logistic regression analysis showed that only δMPT is an indicator regarding the need for an additional procedure in patients who underwent SPA (*p* = 0.039, OR = 0.831). That is, patients who have a large improvement of MPT are significantly less likely to undergo an additional procedure.

## Discussion

The initial concept of SPA for voice improvement emerged, almost 25 years ago, in a young male adult with a hemilaryngeal immobility, presenting with dyspnea on effort, referred otalgia and dysphonia. Laryngostroboscopic examination revealed a posterior glottic insufficiency and a mechanical ulcer on the anteromedially prolapsed immobile arytenoid, where it collided with the contralateral mobile arytenoid. Following SPA not only resolved his dyspnea and ulcer, but surprisingly also his voice and the glottic closure improved significantly. Since then, senior author HM has been performing SPA for voice improvement in patients with unilateral hemilaryngeal immobility, as well as in patients with normal bilateral laryngeal mobility, presenting with an incomplete posterior glottic closure apparently due to an obstructing arytenoid.

However, sometimes contralateral hemilaryngeal compensation took several months to develop, during which period the patient’s voice remained inadequate. Therefore, to reduce the period of dysphonia, while awaiting contralateral compensation following SPA, concomitant injection augmentation is routinely performed since 2011.

### Voice outcomes

Our results showed significant improvement in both VHI and MPT in patients who underwent SPA, both with and without concomitant injection augmentation. Short-term voice outcomes (within 6 months after procedure), in terms of both VHI and MPT, were significantly better in patients who received injection augmentation concomitantly to SPA, reducing the period of severe dysphonia. Furthermore, concomitant injection augmentation significantly improved long-term MPT in patients who underwent SPA.

Voice improvement after SPA, both in terms of MPT as well as VHI, may seem modest as compared to some favorable outcomes reported for other phonosurgical procedures addressing anterior glottic insufficiency such as e.g. medialization thyroplasty and injection augmentation, whereas, in contrast, SPA primarily addresses posterior glottic insufficiency, which has proven to be more difficult to correct [13–16]. Furthermore, in our population, there was a large heterogeneity including patients with comprehensive comorbid conditions, such as head and neck irradiation, laryngeal trauma and failed previous phonosurgical procedures, which may influence postoperative voice outcomes.

The correlation between a higher pre-operative VHI and better postoperative *δ*VHI outcomes is, in our opinion, of no clinical consequence. This observation may be explained by the “statistical floor effect”, which arises from the fact that the amount of possible recovery is related to initial severity of presentation. That is, patients with VHI of 40 can only recover (approach VHI 0) by that amount, while patients with VHI of 80 can recover ‘twice as much’ [17].

Even though 43% of our population underwent injection augmentation concomitantly to SPA, our results show that patients who did not undergo concomitant injection augmentation also had significantly improved short- and long-term voice outcomes postoperatively. Moreover, both *δ*VHI > 6 months and *δ*MPT > 6 months outcomes showed no significant improvement of voice outcomes in patients who underwent concomitant injection augmentation as compared to SPA only. Besides, it is expected that postoperative long-term voice improvement is a consequence of adequate contralateral hemilaryngeal compensation, facilitated by SPA, more than of concomitant injection augmentation, since with time the injection material used for injection augmentation dissipates [18].

Accordingly, our results show that SPA alone can result in voice improvement in patients with posterior glottic insufficiency due to an obstructing anteromedially prolapsed arytenoid. Moreover, laboratory experiments on excised human larynges, by Enoki et al. [19] (personal communication), in which arytenoid positional asymmetry was simulated, showed incomplete posterior glottic closure in all ten larynges as a result of superomedial contact between the arytenoids. A median increase in the distance between the vocal processes of 1.74 mm was found, providing experimental evidence of this clinical condition and the rationale for SPA.

Ultimately, it is our considered opinion that benefits from SPA regarding the voice are expected to be long-lasting, even after the injected material of concomitant injection augmentation has been resorbed. Nevertheless, our results underline the benefits of concomitant injection augmentation in early postoperative voice improvement following SPA, while gradually contralateral compensation facilitated by SPA can develop.

### Complications

Most observed postoperative complications were regarded as minor (Clavien–Dindo Grade I [12]) and often recovered spontaneously. No long-term aspiration problems or dysphagia were observed. Only in three patients, antibiotics were prescribed and in two patients, an additional procedure was required to remove persistent granuloma (Clavien–Dindo Grade II/III [12]). It is worth noting that postoperative dyspnea, laryngeal edema and a sore throat only occurred in patients who underwent concomitant injection augmentation. These complications have been described in the literature after injection augmentation only [19]. Therefore, it is uncertain whether these complications should be attributed to SPA, to the injection augmentation, or specifically to the combined procedure.

In an effort to reduce the risk of postoperative granuloma, the overlying mucosa was preserved whenever possible. Although we did not specifically record the condition of the overlying mucosa in every case, it is estimated that, due to the low laser energy settings and regularly intermittent inspection, the overlying mucosa was preserved in the vast majority of the patients.

### Additional procedures

Low *δ*MPT was a significant indicator regarding additional procedures following SPA. However, concomitant injection augmentation, even though significantly correlated to *δ*MPT outcome, was no significant explaining factor for not requiring an additional procedure in our population. Therefore, we presume that injection augmentation concomitantly to SPA has no effect on the need for an additional procedure.

As previously pointed out, adequate contralateral hemilaryngeal compensation cannot be fully guaranteed to occur following SPA and has to be awaited. Should compensation not occur after SPA, an additional procedure can still be safely performed, without SPA interfering with the potential final outcome.

Ultimately, 68% of patients who underwent SPA required no additional procedure and were satisfied with their postoperative voice outcome. Patients requesting an additional procedure for further voice improvement can obviously be considered a disappointing outcome of SPA. However, it is important to realize that all patients presented with posterior glottic insufficiency and that most patients were initially referred for (comprehensive) laryngeal framework surgery, mostly including arytenoid management. All patients who underwent SPA still remain amenable for such laryngeal framework procedures. Not only were laryngeal framework procedures avoided in 81% of our population, but also was additional arytenoid adduction only required in four patients, underlining the effect of SPA on improvement of a posterior glottic insufficiency in most patients.

### Therapeutic options for posterior glottic insufficiency

Posterior glottic insufficiency can be challenging to address. Well-known therapeutic options are injection augmentation [20, 21] and medialization thyroplasty [22], performed with or without arytenoid adduction [23] or arytenopexy [24]. However, injection augmentation or medialization thyroplasty are better suited to correct more anterior located glottic insufficiencies, rather than the more difficult to correct posterior glottic insufficiencies [13–16]. Although arytenoid adduction or arytenopexy have proven their value to correct posterior glottic insufficiency in patients with unilateral hemilaryngeal immobility, such procedures addressing the arytenoid’s position can be challenging to perform and will not be performed, while there is still chance of spontaneous recovery, during which period (approximately 9 months) dysphonia persists [25]. Furthermore, they cannot be performed in cases with mobile vocal folds without sacrificing arytenoid mobility or in cases of crico-arytenoid fixation.

More recently, non-selective laryngeal reinnervation has become a potential alternative procedure for voice improvement in selected patients with hemilaryngeal immobility [26]. But this procedure, just like laryngeal framework surgery, also requires neck surgery and will not be taken into consideration as long as spontaneous recovery can still occur.

In contrast to laryngeal framework surgery and non-selective laryngeal reinnervation, SPA is a simpler, less invasive procedure in patients where the posterior glottic insufficiency seems to be the result of arytenoid obstruction.

Besides, since SPA does not interfere with potential spontaneous recovery of function, it can be performed shortly after the voice disorder’s occurrence as opposed to arytenopexy, arytenoid adduction or non-selective laryngeal reinnervation. The period of dysphonia can thus be reduced significantly in patients who are amenable for SPA. In addition, patients with crico-arytenoid fixation and patients with mobile vocal folds are also eligible for SPA in cases with arytenoid obstruction. Furthermore, SPA requires only brief hospital admission and short surgical time, and can be performed under general anesthesia. Moreover, in patients with comprehensive comorbidities (e.g. irradiated neck, laryngeal trauma or previously failed phonosurgical procedures), SPA can easily be performed. Besides, if voice outcomes after SPA are inadequate, most patients are still amenable for aforementioned therapeutic options without interfering with their potential final outcome.

### Limitations

There are some limitations of the presented study, which need to be addressed in future research.

First, all surgical procedures and follow-up consultations were performed by a single surgeon. Even though this will diminish inter-patient variability regarding surgical procedure and eradicate bias concerning treatment and evaluation, it is beyond dispute that, as a consequence, our results are susceptible to bias.

Second, because of the retrospective nature of this study, voice outcomes between patients who only underwent SPA and patients who underwent SPA including concomitant injection augmentation cannot easily be compared.

Furthermore, a recent meta-analysis showed that there is compelling evidence that injection augmentation within 6 months following the onset of unilateral vocal fold paralysis, decreases the rate of medialization thyroplasty [27].

Although injection augmentation and medialization thyroplasty generally address anterior glottic insufficiencies rather than posterior glottic insufficiencies, which is the primary indication of SPA, it may very well be that also early performance of SPA with or without concomitant injection augmentation may further reduce the need for laryngeal framework surgery.

The positive effect of early injection augmentation on the reduced rate of medialization thyroplasty can, however, not explain the reduction of required laryngeal framework procedures in our population, since the mean time elapsed between the onset of hemilaryngeal immobility and SPA was 18 months (ranging from 3 months until more than 50 years). Nevertheless, the authors would like to advocate the performance of SPA in combination with injection augmentation early following the onset of hemilaryngeal immobility, as has become our practice during the last years. The relatively long period between onset of hemilaryngeal immobility and SPA in our series reflects the tertiary referral position of our center, where patients are sometimes sent rather late as “last resort”.

Another limitation of our study is that one of our primary outcomes is VHI, which is subjective and, therefore, susceptible to biased assessment, particularly in unblinded studies [28]. Even though our other primary outcome (MPT) is a more objective measure, other objective measures (e.g. size and documentation of glottal gap [16]) could be included in further studies.

A final problem encountered in our study proved to be the long-term follow-up, since patients who were satisfied with their voice result tended to be less likely to turn up for follow-up consultation.

Unfortunately, some of the addressed challenges are inherent to any retrospective study and further prospective research with systematic long-term follow-up is warranted to accurately assess the efficacy of SPA, especially in comparison to other phonosurgical procedures.

## Conclusion and recommendations

Superomedial partial arytenoidectomy (SPA) is a safe and efficient procedure for voice improvement in patients with posterior glottic insufficiency due to an obstructing anteromedially prolapsed arytenoid. In those patients amenable for SPA, comprehensive and challenging procedures for correction of posterior glottic insufficiency may be avoided in many patients. The authors recommend performing this procedure in combination with injection augmentation, in patients with hemilaryngeal immobility, as a single-stage procedure, so that compensation can progress gradually and voice improvement is instantaneous.

Prospective research is needed to accurately assess the efficacy of SPA, compared to other procedures for voice improvement, in patients with posterior glottic insufficiency.
